# Clinical characteristics, antimicrobial susceptibility patterns, and treatment outcomes of nontuberculous mycobacterial diseases in eastern China: a retrospective cohort study (2019–2022)

**DOI:** 10.1128/spectrum.01374-25

**Published:** 2025-12-09

**Authors:** Huijie Wang, Haibo Hua, Yuanyuan Chen, Qiang Zhan, Huaqiong Huang

**Affiliations:** 1 Tuberculosis Department, Zhejiang Hospital of Integrated Traditional Chinese and Western Medicine, Hangzhou, Zhejiang, China; 2 Key Laboratory of Respiratory Disease of Zhejiang Province, Department of Respiratory and Critical Care Medicine, Second Affiliated Hospital of Zhejiang University School of Medicine 89681, Hangzhou, Zhejiang, China; Assistance Publique - Hopitaux de Paris Universite Paris Saclay, Clamart, France

**Keywords:** nontuberculous mycobacteria, NTM clinical characteristics, NTM species identification, **a**ntimicrobial susceptibility test

## Abstract

Nontuberculous mycobacteria (NTM) are opportunistic pathogens, and the global incidence of NTM diseases has been steadily increasing, posing significant public health challenges. This study retrospectively analyzed clinical data from 136 cases of NTM disease diagnosed at the Zhejiang Provincial Tuberculosis Diagnosis and Treatment Center between 2019 and 2022. It was observed that NTM diseases in this region predominantly affected middle-aged and elderly men, with chronic cough being the principal symptom (74.3%). Bronchiectasis (57.4%) and cavities (38.2%) were the main imaging characteristics. The results of species identification demonstrated that *Mycobacterium intracellulare* (62.5%) was the dominant strain. Antimicrobial susceptibility testing revealed that *Mycobacterium avium* complex exhibited relatively favorable sensitivity to clarithromycin, amikacin, ethambutol, rifapentine, rifampicin, and moxifloxacin, while the *Mycobacterium chelonae/Mycobacterium abscessus* complex showed sensitivity only to amikacin (66.7%). In total, 66.2% of patients received anti-NTM therapy. Patients completing ≥6 months of treatment achieved significantly higher efficacy than those with <6 months (81.82% vs 57.14%, *P* < 0.01). The study highlights that NTM diseases in this region are predominantly caused by *M. intracellulare*, with characteristic clinical/imaging features aiding diagnosis. Treatment regimens guided by susceptibility testing and maintained for ≥6 months significantly improve outcomes, emphasizing the importance of species identification and standardized therapeutic duration in NTM management. These findings provide evidence-based insights for optimizing regional NTM diagnosis and treatment strategies.

**IMPORTANCE:**

Nontuberculous mycobacteria (NTM) infections are rising globally, but their characteristics vary by region, complicating diagnosis and treatment. This study addresses a critical gap by analyzing 136 cases from Eastern China, providing the first detailed insights into local NTM disease patterns. By linking pathogen types, drug resistance, and treatment duration to outcomes, this work equips doctors in Eastern China to diagnose faster, choose effective therapies, and improve patient survival. These findings also urge policymakers to prioritize localized data collection and standardized protocols for NTM management globally.

## INTRODUCTION

Nontuberculous mycobacteria (NTM) are widely distributed in nature. According to their growth rates, they can be divided into two major categories: rapidly growing mycobacteria (RGM) and slowly growing mycobacteria (SGM) (1). In recent years, with the increasing attention to NTM and the development of molecular biology, people’s understanding of NTM has been gradually deepening. So far, more than 200 species and various subspecies have been found (2). NTM has very close contact with humans. It can be encountered in natural water systems, municipal water distribution systems, and soil (3), and the infections caused by it have received more and more attention.

According to investigations in recent years, the incidence of NTM diseases in most regions around the world has shown a significant upward trend (4, 5), and it has also increased in China. The epidemiological investigations of tuberculosis over the years have shown that the isolation rate of NTM increased from 4.3% in 1979 to 22.9% in 2010 (6). The authors of a study collected 4,917 mycobacterium culture-positive strains from 72 tuberculosis monitoring sites in 31 provinces in 2013 and identified 317 NTM strains, accounting for 6.4% (7). Globally, the most common NTM pathogen is the *Mycobacterium avium* complex (MAC), which includes six slowly growing mycobacteria such as *Mycobacterium avium* and *Mycobacterium intracellulare*, although the prevalence varies according to geographical regions, gender, and age (8). The *Mycobacterium abscessus* complex (MABC) has a very high antibiotic resistance, and the incidence of diseases caused by this strain is also increasing year by year, which is an increasingly serious problem for East Asian countries, including Japan, South Korea, Taiwan, and China (9). Now, NTM has seriously affected public health, and there are more and more related reports. This may be related to the aging of the population, the increase in the number of immunocompromised people, the improvement of detection methods, and the enhanced awareness of NTM diseases among clinicians (5, 10, 11).

Different types of NTM have different drug sensitivities, and there is uncertainty in each NTM’s drug sensitivity. Therefore, the treatment cannot refer to that of tuberculosis. Thus, both the “NTM Disease Treatment Guidelines” of the American Thoracic Society in 2020 (1) and the “Diagnosis and Treatment Guidelines for Non-tuberculous Mycobacterial Diseases” in China (6) pointed out that the results of species identification and antimicrobial susceptibility tests (AST) are very important before treating NTM diseases. Although there are controversies regarding *in vitro* drug susceptibility tests for NTM because of the differences from clinical results (12), currently, according to the standardized protocol of the Clinical and Laboratory Standards Institute (CLSI) (13), the broth microdilution method is used for drug susceptibility tests. In slowly growing mycobacteria, clear correlations have been established for macrolides and amikacin in MAC pulmonary diseases and for rifampin in *Mycobacterium kansasii* pulmonary diseases (14, 15), and clarithromycin is recommended as the macrolide drug for detection. For RGM, the recommended drugs for testing are amikacin, cefoxitin, ciprofloxacin, clarithromycin, doxycycline or minocycline, imipenem, linezolid, moxifloxacin, trimethoprim-sulfamethoxazole, and tobramycin (16). AST plays an effective guiding role in the clinical treatment of NTM diseases.

In recent years, although great progress has been made in the pathogenesis, epidemiology, and diagnostic methods of NTM diseases and the level of AST, the treatment effect of NTM diseases is still not satisfactory. Studies have shown that the effective treatment rate for MAC pulmonary diseases is between 32% and 65%, and 12%–16% of the enrolled patients did not complete the treatment (17–21). The effective treatment rate for *Mycobacterium abscessus* pulmonary diseases is only 34%, and it even drops to 20% for refractory diseases, with a high recurrence rate (20). The main reasons for treatment failure may include long treatment courses, significant drug side effects, and reinfection caused by environmental factors (17). NTM diseases have clearly become a complex and severe public health problem. At present, there are few treatment studies on NTM diseases in China, and the overall clinical treatment results are not optimistic.

This study retrospectively analyzed the cases of NTM diseases diagnosed in the Tuberculosis Diagnosis and Treatment Center of Zhejiang Province in the past 3 years with complete strain identification and minimal inhibitory concentration (MIC) data, obtained the characteristics of strain types and MICs, and simultaneously analyzed the relevant clinical data of the cases, to provide a scientific basis for formulating treatment plans and conducting treatment follow-up for NTM in this region in the future.

## MATERIALS AND METHODS

### Data acquisition

**Fig 1:**
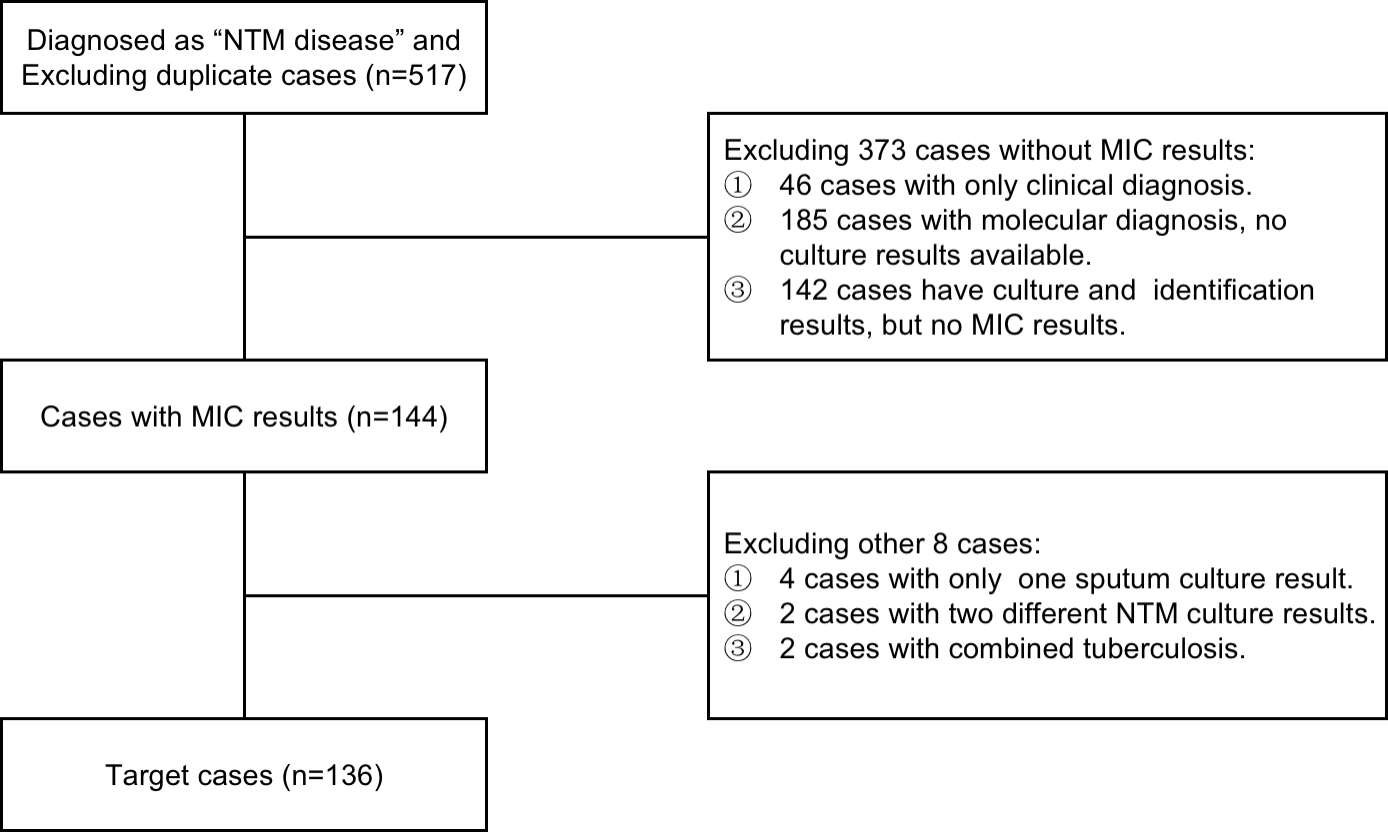
Flowchart of case selection process. According to the inclusion and exclusion criteria, 517 cases diagnosed with NTM disease were screened. Finally, 136 cases with confirmed mycobacterial culture, species identification, and antimicrobial susceptibility testing results were analyzed.

We conducted a retrospective observational study of 517 patients diagnosed with NTM diseases at the Tuberculosis Diagnosis and Treatment Center of Zhejiang Province, Zhejiang Hospital of Integrated Traditional Chinese and Western Medicine from July 2019 to July 2022 (Fig. 1). Referring to the “2020 Diagnosis and Treatment Guidelines for Non-tuberculous Mycobacterial Disease” (6), this study screened cases that met the diagnostic criteria and had bacterial type identification and MIC test results. The exclusion criteria encompass patients whose lesion site specimens fail to meet the bacterial testing criteria stipulated in the NTM treatment guidelines, as well as those with concurrent active pulmonary tuberculosis.

The demographic and clinical data of patients were retrieved from the Electronic Medical Record System. The collected variables encompass gender, age, comorbidities, body mass index (BMI), clinical manifestation, lung CT imaging features, and laboratory test results, including T-SPOT-TB, sputum smear for acid-fast bacilli detection, metagenomics next-generation sequencing (mNGS), serum albumin levels, species identification, and AST results. Clinical data should be collected from patients who have received treatment for over 2 months, with the 6th month of treatment as the observation point. Telephone follow-up information must be documented for patients failing to return for follow-up. Due to the low positive rate of sputum smear results, some outpatient patients did not undergo sputum smear examinations, and some patients were being treated at other hospitals. Accurate sputum smear examination results could not be effectively obtained. Hence, post-treatment sputum smear results were not included in the statistical analysis.

### Identification of NTM isolates and antimicrobial susceptibility testing

Processed samples were inoculated into MGIT culture tubes and subsequently placed in the BACTEC MGIT 960 system for culturing. For positive cultures, MPB64 detection is conducted to differentiate between *Mycobacterium tuberculosis* and non-*Mycobacterium tuberculosis*. The Extractor 36 Nucleic Acid Rapid Extraction Instrument (CapitalBio, Chengdu, China) was utilized for DNA extraction from mycobacterial cultures obtained via the BACTEC MGIT 960 system. PCR amplification of the extracted DNA was performed, followed by hybridization with the HybSet Gene Microarray Hybridization Kit (CapitalBio, Chengdu, China). Signal detection and result interpretation employed the LuxScan 10K/B Scanner (CapitalBio) and its proprietary software.

The determination of the MIC using the mycobacterial susceptibility testing kit (Encode, Zhuhai, China) was performed as per the manufacturer’s instructions. NTM growth in the wells treated with drugs was assessed in comparison to the NTM growth in a drug-free control well on days 3–7. The MIC of a drug refers to the lowest concentration that can inhibit the visible growth of a microorganism in a well. The resistance breakpoints were based on Clinical Laboratory Standards Institute (CLSI M24S (13)). If CLSI does not provide a drug sensitivity breakpoint for a certain NTM, then refer to the breakpoint interpretation results of other NTMs in the same subgroup and record the MIC value accurately.

### Statistical analysis

Data were analyzed using IBM SPSS Statistics 25 (Armonk, NY: IBM Corp.). Continuous variables that adhered to a normal distribution and demonstrated homogeneity of variance were presented as mean ± standard deviation. Statistical analyses of these variables were conducted using the Student’s t-test. Categorical variables were summarized as frequencies and percentages and subsequently subjected to statistical analysis using the chi-square test. Notably, for samples with theoretical frequencies below 5, the corrected chi-square test was applied, whereas for those with theoretical frequencies less than 1, Fisher’s exact probability method was utilized. Statistical results were deemed significant when the *P*-value was less than 0.05.

## RESULTS

### Demographic and clinical data

According to the inclusion and exclusion criteria in the method, we finally retrospectively analyzed 136 cases of NTM disease in the Tuberculosis Diagnosis and Treatment Center of Zhejiang Province from 2019 to 2022 (Table 1). The average age of the 136 patients with NTM disease was 62.91 ± 12.23 years. There were 85 males and 51 females, with a male-to-female ratio of 1.67:1. 25.7% (35/136) of patients had a history of tuberculosis, and the tuberculosis was stable within 2 years without anti-tuberculosis treatment. Among all patients admitted, 54.4% (74/136) had a BMI (Kg/m^2^) below 18.5, 41.2% (56/136) had a BMI between 18.5 and 23.9, and 4.4% (6/136) had a BMI above 23.9. Furthermore, we analyzed the comorbidities and found that those with a history of bronchiectasis were the most common (56.6%, 77/136), followed by chronic obstructive pulmonary disease (COPD) (20.6%, 28/136). Chronic cough was the most common clinical manifestation in 136 patients with NTM disease, accounting for 74.3% (101/136).

**TABLE 1:** Demographic and clinical characteristics of the study population (*n* = 136)

Characteristic	Value
General information	
Sex (male/female)	85/51
Age (years)	62.91 years
BMI (kg/m^2^)	Male: 18.10±2.99; female: 18.79±3.29
With a history of tuberculosis	35
Comorbidities	*n* (%)
Bronchiectasis	77 (56.6)
Chronic obstructive pulmonary disease	28 (20.6)
Hypertension	15 (11.0)
Chronic stomach disease	14 (10.3)
Type 2 diabetes	14 (10.3)
Rheumatic immune disease^*a*^	13 (9.5)
Heart disease	12 (8.9)
Leukopenia	8 (5.9)
Thrombocytopenia	6 (4.4)
Cirrhosis	6 (4.4)
Renal insufficiency	6 (4.4)
Malignant tumors	5 (3.7)
Silicosis	4 (2.9)
Interstitial pneumonia	3 (2.2)
Mental illness	2 (1.5)
Asthma	1 (0.7)
Femoral head necrosis	1 (0.7)
Allergic dermatitis	1 (0.7)
Acquired immune deficiency syndrome	1 (0.7)
Clinical manifestation	
Chronic cough	101 (74.3)
Hemoptysis	31 (22.8)
Shortness of breath	30 (22)
Fever	13 (9.6)
Asymptomatic patients with lung shadows	13 (9.6)
Fatigue	6 (4.4)
Chest pain	6 (4.4)
Emaciation	2 (1.5)
Joint pain	2 (1.5)
Others^*b*^	3 (2.2)
Laboratory examination	
Serum albumin levels	
<25 g/L	2 (0.16)
25–35 g/L	67 (49.2)
>35 g/L	67 (49.2)
T-SPOT-TB	
Positive	15 (11)
Negative	121 (89)
Acid-fast bacilli in sputum smear	
Positive	44 (32.4)
Negative	92 (67.6)
Manifestations of lung CT images	
Bronchiectasis	78 (57.4)
Cavitation	52 (38.2)
Patchy and streaky shadows	38 (27.9)
Nodules	17 (12.5)
Emphysema	17 (12.5)
Exudation	15 (11)
Local pleural lesions	14 (10.3)
Partial lung destruction	13 (9.6)
Miliary nodule-like changes	1 (0.8)

^*a*^ Rheumatoid immune diseases include rheumatoid arthritis, systemic lupus erythematosus, and ankylosing spondylitis.

^*b*^ The other three cases include two patients with lower back pain and one patient with a finger wound that failed to heal after a long time.

Statistical analysis of laboratory examination outcomes from 136 NTM patients revealed that the majority maintained serum albumin levels within a moderately high range. Regarding the T-SPOT-TB test, 11% (15/136) of the enrolled cases yielded a positive result, whereas negative cases constituted 89% (121/136). Notably, the acid-fast bacilli smear positivity rate among all NTM patients was merely 32.4% (44/136). Chest CT imaging in NTM patients revealed diverse manifestations, with bronchiectasis being the predominant finding (57.4%, 78/136), followed by cavities (38.2%, 52/136) (particularly thin-walled cavities). Other observed features included patchy and streaky shadows, nodules, emphysema, etc.

### Species identification

Among the 136 NTM cases, the main source of specimens submitted for examination was bronchoalveolar lavage fluid, which constituted 89.0% (121/136 cases). The proportion of sputum samples submitted more than twice was 8.8% (12/136), and tissue accounted for 2.2% (3/136). Regarding the results of bacterial strain identification, *M. intracellulare* accounted for 62.5% (85/136). The *Mycobacterium chelonae/*MABC accounted for 16.2% (22/136). *M. avium* made up 14.7% (20/136). *M. kansasii* accounted for 5.1% (7/136), and *Mycobacterium xenopi* accounted for 1.5% (2/136).

The research also discovered that within the 136 cases, 25.7% (35/136) had the bronchoalveolar lavage fluid sent for mNGS testing. The outcomes indicated that 30 of them were in line with the culture identification results, attaining an accuracy rate of 85.7% (30/35), and 11.4% (4/35) presented negative results. In one specimen, the mNGS indicated *M. kansasii*, yet the bacterial strain identification result was *M. intracellulare*.

### Antimicrobial susceptibility testing

Among the 136 NTM strains, 85 were *M. intracellulare*, and we analyzed their drug susceptibility results. CLSI has provided four drugs with definite antimicrobial susceptibility breakpoints for MAC, namely the first-line drugs clarithromycin and amikacin and the second-line drugs moxifloxacin and linezolid (13). The data in this study show that *M. intracellulare* has a susceptibility rate of 98.8% (84/85) to the two first-line drugs. It also has a susceptibility rate of 71.8% (61/85) to the second-line drug moxifloxacin. Its susceptibility to linezolid was relatively poor, with a susceptibility rate of 40% (34/85) and a resistance rate as high as 60% (51/85). Among the drugs for which no antimicrobial susceptibility breakpoints were given, the proportion of *M. intracellulare* with an MIC of ethambutol ≤2.5 µg/mL was 81.2% (69/85), indicating relatively good *in vitro* susceptibility. The proportion of *M. intracellulare* with an MIC of rifampicin ≤1 µg/mL was 58.8% (50/85), and rifapentine ≤0.5 µg/mL was 74.1% (63/85), also showing good susceptibility. Most strains of *M. intracellulare* were resistant to doxycycline, tobramycin, and imipenem, with the resistance rates being 91.8%, 84.7%, and 100%, respectively (Table 2).

**TABLE 2:** Antimicrobial susceptibility testing results of 136 NTM strains (n [%])^*a*^

	*M. intracellulare* (*n* = 85)			*M. avium* (*n* = 20)			*M. chelonae/* *M. abscessus* (*n* = 22)			*M. kansasii* (*n* = 7)			*M. xenopi* (*n* = 2)		
Clarithromycin	S	I	R	S	I	R	S	I	R	S	I	R	S	I	R
	84 (98.8)	0	1 (1.2)	20 (100)	0	0	8 (36.4)	6 (27.3)	8 (36.4)	7 (100)	0	0	2 (100)	0	0
Amikacin	S	I	R	S	I	R	S	I	R	S	I	R	S	I	R
	84 (98.8)	0	1 (1.2)	19 (95)	0	1 (5)	20 (90.9)	0	2 (9.1)	7 (100)	0	0	2 (100)	0	0
Moxifloxacin	S	I	R	S	I	R	S	I	R	S	I	R	S	I	R
	61 (71.8)	17 (20.0)	7 (8.2)	18 (90)	2 (10)	0	4 (18.2)	6 (27.3)	12 (54.5)	6 (85.7)	1 (14.3)	0	2 (100)	0	0
Linezolid	S	I	R	S	I	R	S	I	R	S	I	R	S	I	R
	34 (40.0)	0	51 (60.0)	8 (40)	0	12 (60)	13 (59.1)	0	9 (40.9)	6 (85.7)	0	1 (14.3)	2 (100)	0	0
Ethambutol	≤2.5	5–10	≥20	≤2.5	5–10	≥20	≤2.5	5–10	≥20	≤2.5	5–10	≥20	≤2.5	5–10	≥20
	69 (81.2)	8 (9.4)	8 (9.4)	18 (90)	1 (5)	1 (5)	3 (13.6)	3 (13.6)	16 (72.7)	3 (42.9)	4 (57.1)	0	1 (50)	1 (50)	0
Cefoxitin	≤16	32–64	≥64	≤16	32–64	≥64	S	I	R	≤16	32–64	≥64	≤16	32–64	≥64
	47 (55.5)	31 (36.5)	7 (8.2)	3 (15)	16 (80)	1 (5)	8 (36.4)	13 (59.1)	1 (4.5)	0	0	7 (100)	0	1 (50)	1 (50)
Rifampicin	≤1	4	≥16	≤1	4	≥16	≤1	4	≥16	S	I	R	S	I	R
	50 (58.8)	28 (32.9)	7 (8.2)	8 (40)	5 (25)	7 (35)	14 (63.6)	5 (22.7)	3 (13.6)	5 (71.4)	1 (14.3)	1 (14.3)	2 (100)	0	0
Doxycycline	≤1	2–4	≥8	≤1	2–4	≥8	S	I	R	S	I	R	S	I	R
	1 (1.2)	6 (7.1)	78 (91.8)	1 (5)	3 (15)	16 (80)	1 (4.5)	1 (4.5)	20 (90.9)	0	0	7 (100)	0	0	2 (100)
Tobramycin	≤2	4	≥8	≤2	4	≥8	S	I	R	≤2	4	≥8	≤2	4	≥8
	2 (2.4)	11 (12.9)	72 (84.7)	2 (10)	4 (20)	14 (70)	1 (4.5)	0	21 (95.5)	0	0	7 (100)	0	2 (100)	0
Imipenem	≤4	8–16	≥32	≤4	8–16	≥32	S	I	R	≤4	8–16	≥32	≤4	8–16	≥32
	0	0	85 (100)	0	0	20 (100)	0	0	22 (100)	0	0	7 (100)	0	0	2 (100)
Rifapentine	≤0.5	–^*b*^	≥2	≤0.5	–	≥2	≤0.5	–	≥2	≤0.5	–	≥2	≤0.5	–	≥2
	63 (74.1)	–	22 (25.9)	16 (80)	–	4 (20)	1 (4.5)	–	21 (95.5)	0	–	7 (100)	1 (50)	–	1 (50)
Azithromycin	≤4	16	≥32	≤4	16	≥32	≤4	16	≥32	≤4	16	≥32	≤4	16	≥32
	23 (27.1)	44 (51.8)	18 (21.2)	4 (20)	11 (55)	5 (25)	3 (13.6)	15 (68.2)	4 (18.2)	3 (42.9)	4 (57.1)	0	1 (50)	1 (50)	0
Sulfamethoxazole	≤8	16–80	>80	≤8	16–80	>80	S	I	R	S	I	R	S	I	R
	0	33 (38.8)	52 (61.2)	1 (5)	12 (60)	7 (35)	5 (22.7)	–	17 (77.3)	3 (42.9)	0	4 (57.1)	1 (50)	0	1 (50)
Minocycline	<16	16–64	>64	<16	16–64	>64	<16	16–64	>64	<16	16–64	>64	S	I	R
	17 (20)	53 (62.4)	15 (17.6)	5 (25)	13 (65)	2 (10)	2 (9.1)	8 (36.4)	12 (54.5)	2 (28.6)	5 (71.4)	0	0	0	2 (100)
Gatifloxacin	<1	1–4	≥8	<1	1–4	≥8	<1	1–4	≥8	<1	1–4	≥8	<1	1–4	≥8
	21 (24.7)	60 (70.6)	4 (4.7)	11 (55)	9 (45)	0	1 (4.5)	17 (77.3)	4 (18.2)	5 (71.4)	2 (28.6)	0	2 (100)	0	0

^*a*^ *M. intracellulare, Mycobacterium intracellulare*; *M. avium, Mycobacterium avium*; *M. chelonae/M. abscessus*, *Mycobacterium chelonae/Mycobacterium abscessus *complex; *M. kansasii*, *Mycobacterium kansasii; M. xenopi*, *Mycobacterium xenopi*; S, susceptible; I, intermediate; R, resistant. The unit of MIC of a drug is μg/mL.

^*b*^ – indicates not applicable (N/A); the drug does not have an intermediate value, and the corresponding number of strains is zero.

Twenty strains were *M. avium*. Its sensitivity closely matched that of *M. intracellulare*. The susceptibility rates to clarithromycin and amikacin were 100% (20/20) and 95% (19/20), respectively. Its sensitivity to moxifloxacin surpassed that of *M. intracellulare*, reaching 90% (18/20). The sensitivity to linezolid was also less than satisfactory. Regarding drugs without defined breakpoints, the proportion of *M. avium* with an MIC of ethambutol ≤2.5 µg/mL was 90% (18/20), rifapentine ≤0.5 µg/mL was 80% (16/20), rifampicin ≤1 µg/mL was 40% (8/20), and gatifloxacin ≤1 µg/mL was 55% (11/20) (Table 2).

Twenty-two strains were *M. chelonae/*MABC. CLSI recommends performing drug susceptibility tests for *M. chelonae/*MABC with clarithromycin, amikacin, moxifloxacin, linezolid, cefoxitin, tobramycin, imipenem, and sulfamethoxazole, and providing corresponding breakpoints (13). The data of this study indicated that *M. chelonae/*MABC has relatively good sensitivity to amikacin, with a susceptibility rate reaching 90.9% (20/22). Linezolid comes next, with a susceptibility rate of 59.1% (13/22), but the resistance rate of linezolid also reaches 40.9% (9/22). In *M. chelonae*/MABC, clarithromycin had similar susceptibility (36.4%, 8/22), intermediate (27.2%, 6/22), and resistance (36.4%, 8/22) rates. Azithromycin had 68.2% (15/22) intermediate (MIC = 16 µg/mL). Cefoxitin had 36.4% (8/22) susceptibility, 59.1% (13/22) intermediate, and 0.45% (1/22) resistance. Moxifloxacin had 18.2% (4/22) susceptibility, 27.2% (6/22) intermediate, and 54.6% (12/22) resistance. Doxycycline, tobramycin, imipenem, and sulfamethoxazole had high resistance rates of 90.9% (20/22), 95.4% (21/22), 100% (22/22), and 77.3% (17/22), respectively. For drugs without defined breakpoints, 72.7% (16/22) and 95.5% (21/22) of the complex showed high MIC values for ethambutol and rifapentine, respectively (Table 2).

Among the isolated strains, seven were *M. kansasii*. According to the recommendations of the CLSI, drug susceptibility tests for clarithromycin, rifampicin, amikacin, doxycycline, moxifloxacin, and linezolid should be carried out on *M. kansasii*, along with the specification of corresponding breakpoints (13). This study revealed that all seven cases of *M. kansasii* were 100% sensitive to clarithromycin and amikacin. The sensitivity rates to moxifloxacin and linezolid were 85.7% (6/7), and that to rifampicin was 71.4% (5/7). Regarding sulfamethoxazole, the sensitivity rate was 42.9% (3/7), which was approximately on par with its resistance rate of 57.1% (4/7). Notably, a 100% resistance rate (7/7) was observed for doxycycline. For drugs without defined breakpoints, such as ethambutol, azithromycin, minocycline, and gatifloxacin, the majority of the test results demonstrated relatively small to moderate MIC values. By contrast, imipenem, tobramycin, and rifapentine exhibited high MIC values in all seven cases (100%), suggesting that these drugs are not advisable for the treatment of *M. kansasii* (Table 2).

Drug susceptibility tests for *M. xenopi* are largely similar to *M. kansasii*, as the CLSI recommends. However, an additional breakpoint for minocycline has been included. Although the number of isolated *M. xenopi* samples was rather limited, with only two strains in total, the test results demonstrated that this bacterium exhibited relatively favorable sensitivity to clarithromycin, amikacin, moxifloxacin, linezolid, and rifampicin. On the contrary, it showed resistance to doxycycline and minocycline. For drugs without defined breakpoints, such as tobramycin, imipenem, and rifapentine, the results revealed relatively high MIC values, which implies a certain degree of resistance. By contrast, the MIC value of gatifloxacin was relatively low, indicating a tendency toward sensitivity (Table 2).

### Treatment and prognosis analysis

Among the 136 patients with NTM diseases, 66.2% (90/136) received treatment after diagnosis, while 33.8% (46/136) did not receive treatment following their discharge from the hospital post-diagnosis. Among the patients with *M. intracellulare* and *M. avium* diseases, 70.1% (60/85) and 70% (14/20), respectively, received treatment. The initial treatment regimens focused on clarithromycin or azithromycin, ethambutol, and rifampicin, with some regimens adjusted based on the results of AST. Among the patients diagnosed with *M. chelonae/Mycobacterium abscessus* disease, 40.9% (9/22) underwent treatment. The treatment regimens primarily included cefoxitin injection, amikacin injection, and either clarithromycin or azithromycin, with modifications made according to AST outcomes. For *M. kansasii* disease, 85.7% (6/7) of patients received initial treatment, which uniformly consisted of rifampicin, isoniazid, or clarithromycin, along with ethambutol. In cases of *M. xenopi* disease, 50% (1/2) of patients received treatment, with the regimen comprising clarithromycin, rifampicin, and ethambutol. Upon reviewing the 46 cases that did not receive treatment, it was found that 60.8% (28/46) of these patients requested observation and follow-up, 34.8% (16/46) were unable to tolerate the initial treatment, and 4.3% (2/46) were lost to follow-up.

The prognosis of patients was primarily evaluated based on clinical symptoms and lung CT images. Due to the relatively low positive rate of acid-fast bacilli detected by sputum smear and the fact that some patients did not follow up at our hospital and lacked sputum test results, the detection of acid-fast bacilli by sputum smear was not used as an indicator for prognostic evaluation. Among the 90 patients who underwent anti-NTM treatment, 77.8% (70/90) adhered to the treatment regimen for over 2 months (Table 3). Of these 70 patients, 65 experienced either an improvement in their conditions or remained stable, resulting in a treatment efficacy rate of 92.9% (65/70). Considering a treatment duration of 6 months as the observational milestone, 61.1% (55/90) continued treatment for more than 6 months. Among these 55 patients, 45 showed an improvement in their conditions or maintained stability, resulting in a treatment efficacy rate of 81.82% (45/55). There were 35 patients whose treatment duration was less than 6 months, corresponding to a treatment discontinuation rate of 38.9% (35/90). Of these 35 patients, 57.1% (20/35) either improved their conditions or remained stable. The prognosis of patients who received anti-NTM treatment for more than 6 months was significantly better than that of those who prematurely terminated treatment (81.82% vs 57.14%, *P* < 0.01) (Table 5).

**TABLE 3:** Case numbers at different treatment stages^*a*^

	Initial treatment	Treatment for 2 months		Treatment for 6 months	
		Adhere to treatment	Withdraw medication	Adhere to treatment	Withdraw medication
MAC	74	59 (55)	15 (10)	44 (36)	30 (19)
*M. chelonae*/MABC	9	4 (3)	5 (2)	4 (2)	5 (1)
*M. kansasii*	6	6 (6)	0	6 (6)	0
*M. xenopi*	1	1 (1)	0	1 (1)	0
Total	90	70 (65)	20 (12)	55 (45)	35 (20)


^*a*^ The figures in parentheses are the numbers of cases with improvement in condition or remaining stable.

### Influencing factors for anti-NTM treatment

An analysis was carried out on the “treatment group” encompassing 90 patients and the “non-treatment group” comprising 46 patients (Table 4). No significant disparities were observed between the two groups concerning gender, age, BMI, serum albumin levels, manifestations depicted in chest imaging, as well as comorbidities. Regarding symptoms, patients afflicted with chronic cough demonstrated a greater propensity to embrace treatment (68.9% vs 58.7%, *P* < 0.05). By contrast, no significant differences were noted in terms of dyspnea, hemoptysis, and febrile episodes between the two cohorts. Patients with a positive result for acid-fast bacilli in sputum were more prone to opt for anti-NTM treatment (44.44% vs 8.70%, *P* < 0.001). Among patients infected with diverse mycobacterial strains, those with *M. chelonae/M. abscessus* were more likely to decline anti-NTM treatment (28.26% vs 10.00%, *P* < 0.05). For patients infected with *M. avium/M. intracellulare*, *M. kansasii*, and *M. xenopi*, the numbers of those who received treatment and those who did not were approximately equivalent, without any significant difference.

**TABLE 4:** Analysis of influencing factors for whether to receive anti-NTM treatment

	Treatment group *n* (%)	Non-treatment group *n* (%)	*P* value
Total	90 (100.00)	46 (100.00)	
Sex			
Male	59 (65.56)	26 (56.52)	0.303^*b*^
Female	31 (34.44)	20 (43.48)	
Age (years)	62.07±11.79	64.57±13.15	0.263^*a*^
BMI (kg/m^2^)	18.31±3.12	18.44±3.18	0.824^*a*^
Comorbidities			
Bronchiectasis	50 (55.56)	39 (84.8)	0.727^*b*^
Type 2 diabetes	12 (13.33)	2 (4.35)	0.182^*c*^
Renal insufficiency	2 (2.22)	4 (8.70)	0.194^*c*^
Hypertension	9 (10.00)	6 (13.04)	0.592^*b*^
Chronic stomach disease	13 (14.44)	1 (2.17)	0.054^*c*^
Chronic obstructive pulmonary disease	18 (20.00)	10 (21.74)	0.812^*b*^
Clinical manifestation			
Chronic cough	62 (68.9)	39 (58.70)	0.045^*b*^
Shortness of breath	24 (26.7)	6 (13.04)	0.070^*b*^
Hemoptysis	22 (24.4)	9 (19.56)	0.521^*c*^
Fever	6 (6.67)	7 (15.21)	0.109^*b*^
Laboratory examination			
Serum albumin levels (g/L)	35.18±5.23	35.83±7.03	0.543^*b*^
Acid-fast bacilli in a sputum smear (positive)	40 (44.44)	4 (8.70)	<0.001^*b*^
Manifestations of lung CT images			
Bronchiectasis	51 (56.67)	27 (58.70)	0.821^*b*^
Cavitation	37 (41.11)	15 (32.61)	0.334^*b*^
Nodules	11 (12.22)	6 (13.04)	0.891^*b*^
Exudation	10 (11.11)	5 (10.87)	0.966^*b*^
Partial lung destruction	7 (7.78)	6 (13.04)	0.497^*c*^
Local pleural lesions	8 (8.89)	6 (13.04)	0.648^*c*^
Patchy and streaky shadows	24 (26.67)	14 (30.43)	0.643^*b*^
Emphysema	12 (13.33)	5 (10.87)	0.681^*b*^
NTM bacterial types			
MAC	74 (82.22)	31 (67.39)	0.051^*b*^
*M. chelonae/*MABC	9 (40.9)	13 (59.1)	0.006^*b*^
*M. kansasii*	6 (6.67)	1 (2.17)	0.477^*c*^
*M. xenopi*	1 (1.11)	1 (2.17)	1.000^*d*^

^*a*^ Student's t-test.

^*b*^ Chi-square test.

^*c*^ Correction chi-square test.

^*d*^ Fisher's exact probability method.

Taking 6 months as the treatment observation point, an analysis was performed on the “treatment adherence group” and the “treatment termination group” (Table 5). No significant differences were detected between these two groups concerning gender, age, BMI, chest imaging, and mycobacterial strains. Concerning comorbidities, it was found that patients with diabetes mellitus exhibited a relatively higher tendency to struggle with adhering to the treatment regimen (28.57% vs 3.64%, *P* < 0.001). By contrast, no significant distinctions were observed regarding comorbidities such as renal insufficiency, chronic gastric disorders, and hypertension. Moreover, discontinuation of treatment resulting from adverse drug reactions was rather prevalent (57.14% vs 16.36%, *P* < 0.01), particularly the termination of treatment due to gastrointestinal reactions (25.71% vs 3.64%, *P* < 0.001).

**TABLE 5:** Analysis of influencing factors for anti-NTM treatment course and curative effect evaluation

	Treatment adherence group *n* (%)	Treatment termination group *n* (%)	*P* value
Total	55 (100.00)	35 (100.00)	
Sex			
Male	35 (63.64)	24 (68.57)	0.631^*b*^
Female	20 (36.36)	11 (31.43)	
Age (years)	60.18±12.90	65.03±9.22	0.057^*a*^
BMI (kg/m^2^)	18.52±2.97	17.99±3.37	0.429^*a*^
Comorbidities			
Bronchiectasis	15 (27.27)	15 (42.86)	0.034^*b*^
Type 2 diabetes	2 (3.64)	10 (28.57)	<0.001^*c*^
Renal insufficiency	1 (1.82)	1 (2.86)	1.000^*d*^
Hypertension	7 (12.73)	2 (5.71)	0.692^*c*^
Chronic stomach disease	6 (10.91)	7 (20)	0.195^*c*^
Chronic obstructive pulmonary disease	12 (21.82)	6 (17.14)	0.962^*b*^
Acid-fast bacilli in sputum smear (positive)	23 (41.82)	17 (48.57)	0.530^*b*^
Manifestations of lung CT images			
Bronchiectasis	35 (63.64)	16 (45.71)	0.706^*b*^
Cavitation	24 (43.64)	13 (37.14)	0.843^*b*^
Nodules	5 (9.09)	6 (17.14)	0.237^*c*^
Exudation	5 (9.09)	5 (14.29)	0.438^*c*^
Partial lung destruction	5 (9.09)	2 (5.71)	0.914^*c*^
Local pleural lesions	6 (10.91)	2 (5.71)	0.874^*c*^
Patchy and streaky shadows	12 (21.82)	12 (34.29)	0.065^*b*^
Emphysema	8 (14.55)	4 (11.43)	0.778^*c*^
NTM bacterial types			
MAC	44 (80)	30 (85.71)	0.070^*b*^
*M. chelonae/*MABC	4 (7.27)	5 (14.29)	0.288^*c*^
*M. kansasii*	6 (10.91)	0 (0)	0.177^*c*^
*M. xenopi*	1 (1.82)	0 (0)	1.000^*d*^
Adverse reactions	9 (16.36)	20 (57.14)	<0.01^*b*^
Leukopenia	3 (5.45)	4 (11.43)	0.353^*c*^
Abnormal liver function	4 (7.27)	5 (14.29)	0.288^*c*^
Allergic reactions	0 (0)	2 (5.71)	0.159^*d*^
Gastrointestinal reactions	2 (3.64)	9 (25.71)	0.001^*c*^
Controlled NTM	45 (81.82)	20 (57.14)	0.011^*b*^

^*a*^ Student's t-test.

^*b*^ Chi-square test.

^*c*^ Correction chi-square test.

^*d*^ Fisher's exact probability method.

## DISCUSSION

This study incorporated 136 cases of NTM with definite AST results from the Tuberculosis Diagnosis and Treatment Center in Zhejiang Province. A comprehensive retrospective analysis was carried out regarding their clinical features, the outcomes of *in vitro* AST on the strains, as well as the treatment circumstances. It was observed that NTM diseases in this region predominantly affected middle-aged and elderly men, with chronic cough being the principal symptom. Notably, the detection rate of acid-fast bacilli through sputum smear was merely 32.4%. In terms of pulmonary CT manifestations, bronchiectasis and cavities were the main imaging characteristics. The results of strain identification demonstrated that *M. intracellulare* was the most frequently identified strain, followed by the *M. chelonae/*MABC and *M. avium*. Through *in vitro* AST, it was discovered that MAC exhibited relatively favorable sensitivity to clarithromycin, amikacin, and moxifloxacin. By contrast, the *M. chelonae/*MABC was largely insensitive to most drugs, with only a relatively high level of sensitivity to amikacin. Furthermore, we summarized and analyzed the treatment status of the 136 NTM patients. It was found that only 66.2% of these patients opted for anti-NTM drug treatment after diagnosis. Specifically, patients with chronic cough and those with positive acid-fast bacilli in sputum were more inclined to choose anti-NTM treatment. Among patients infected with different strains, those with *Mycobacterium chelonae/M. abscessus* were more likely to decline anti-NTM treatment. Upon further delving into the influencing factors for the course of anti-NTM treatment, it was revealed that diabetic patients often struggled to adhere to the treatment regimen. Moreover, it was quite common for patients to discontinue treatment due to adverse drug reactions, particularly gastrointestinal reactions. Based on the AST results, the disease control in the group that received anti-NTM treatment for 6 months was superior to that in the group with premature termination of treatment.

The clinical manifestations of NTM diseases lack specificity, and misdiagnosis and missed diagnosis are prone to occur in clinical diagnosis. With the increase in the incidence of NTM, more and more studies have summarized the clinical characteristics of NTM diseases. The incidence of NTM diseases increases with age (6, 22), but there are differences in the gender distribution. In Europe (23), Australia, New Zealand (24), and North America (25), the majority of patients are female, while multiple studies in China have shown that males are more commonly affected (26). This may be related to factors such as region, environment, and race to a certain extent. In this study, the majority of NTM cases were male, and the age group of 50–80 years old had a high incidence of NTM, which is consistent with previous studies (10). NTM is an opportunistic pathogen. The risk factors for NTM pulmonary diseases include structural lung diseases such as bronchiectasis, chronic obstructive pulmonary disease, and cystic fibrosis of the lung (27), and extrapulmonary NTM diseases can be caused by skin or tissue damage (28). The cases collected in this study were mainly NTM pulmonary diseases, and the comorbidities were mostly bronchiectasis and chronic obstructive pulmonary disease, which is in line with the above viewpoints. The main manifestations of NTM pulmonary diseases on lung CT are bronchiectasis and thin-walled cavities in the upper lobes (29), and there are certain differences in the CT images compared with those of pulmonary tuberculosis. However, other lung CT manifestations such as nodules, pleural thickening, destroyed lungs, and predilection sites have no significant differences compared with those of pulmonary tuberculosis. This study found that 57.4% of NTM patients showed bronchiectasis on lung CT, and 38.2% showed cavity manifestations, mainly thin-walled cavities, which have indicative significance for clinical diagnosis.

T-SPOT.TB is based on the principle of the release of IFN-γ mediated by T cells after being stimulated by *M. tuberculosis* antigens, and it has a high specificity in diagnosing tuberculosis infection. However, *M. kansasii*, *Mycobacterium marinum*, *Mycobacterium szulgai,* and *Mycobacterium gordonae* among NTM can also show positive results (30). In this study, 121 cases (89%) were negative for T-SPOT.TB, indicating good specificity. In addition to the influence of *M. kansasii* on the positive results, a history of pulmonary tuberculosis in some patients is also an important factor. The etiological examination is helpful for the diagnosis of NTM diseases, but the positive rate of acid-fast bacilli in traditional sputum smear is not high, only 32.3% (44/136). It takes about 1 month to perform mycobacterium culture, identification, and AST on strains using conventional methods, which cannot meet the clinical needs. With the continuous development of molecular biology techniques, the “Guidelines” (6) has included positive results of molecular biology tests in the diagnostic criteria. By means of molecular biology examination methods, it is possible to quickly distinguish between tuberculosis and NTM species types. This study aims to explore the *in vitro* drug susceptibility and treatment of NTM strains, so the cases included in this study are all cases with positive culture results. Among them, 35 cases had bronchoalveolar lavage fluid sent for mNGS testing, and the results of 30 cases were consistent with the culture and identification results, showing a relatively high accuracy rate. Many studies have also shown that mNGS is of great significance for the diagnosis of NTM (31), which can accurately and quickly diagnose NTM. However, at present, due to the high cost of the test, it is difficult to apply it widely.

The distribution of NTM varies in different regions and even at different times. In northern China, slowly growing mycobacteria account for 63.7% of all NTM isolates, while in southern China, this proportion is 53.0% (32). In Beijing and Zhejiang Province, *M. intracellulare* is the predominant species (26, 33). In Shanghai, *M. kansasii* is the dominant species (34). In the Guangzhou area, from 2004 to 2009, the nontuberculous strains were mainly *M. chelonae/abscessus,* but from 2013 to 2016, the *M. avium-intracellulare* complex was shown to be the main NTM (35). A clinical report on NTM pulmonary disease in the Yangtze River Delta region of China showed that *M. intracellulare*, *M. abscessus*, *M. kansasii*, and *M. avium* are the predominant species (36). Among the 136 strains in this study, *M. intracellulare* is the predominant species, accounting for 62.5% (85/136). If counted by the MAC, it accounts for 77.2% (105/136), making it the absolutely dominant strain. Followed by *M. abscessus*, accounting for 16.2% (22/136), while *M. kansasii* and *M. xenopi* are relatively rare.

Most NTM are resistant to first-line anti-NTM drugs, which is related to factors such as the structure of their cell walls and the formation of biofilms (37). Therefore, bacterial identification and AST are crucial before treating NTM disease. (i) MAC is shown to be the dominant species globally and is also the main species causing NTM pulmonary diseases, lymphadenopathies, etc. (38). Clarithromycin is a key drug for the treatment of MAC, and its resistance affects the treatment outcome of MAC. This study shows that MAC is highly sensitive to the first-line drugs clarithromycin and amikacin, and the sensitivity rate to moxifloxacin is also 71.8%–90%, but the sensitivity rate to linezolid is only 40%. Therefore, for the treatment of MAC, clarithromycin, amikacin, and moxifloxacin can be recommended to be empirically included in the treatment regimen. For drugs without clear breakpoints, ethambutol and rifapentine show good sensitivity, but drugs such as doxycycline, tobramycin, imipenem, and minocycline all show high MIC values, indicating a high possibility of resistance. Despite only 40% rifampicin susceptibility in *M. avium* isolates per this study, guidelines (1, 6) retain rifampicin in regimens. Recent studies have reported high rifampicin resistance rates in antimicrobial susceptibility testing (39) and demonstrated that rifampicin neither enhances the antibacterial efficacy of azithromycin-ethambutol dual therapy nor suppresses resistance emergence (40), challenging its role in recommended regimens. Given the unclear correlation between *in vitro* AST results and clinical efficacy, coupled with limited clinical validation even for well-established AST methods, clinicians primarily adhere to guideline recommendations when formulating treatment strategies. The clinical utility of rifampicin against *M. avium* requires further investigation. (ii) Currently, the mycobacterium strain identification kit in our laboratory cannot distinguish between *M. chelonae* and *M. abscessus*. The identification result is the *M. chelonae/*MABC, which is a multidrug-resistant NTM and the pathogenic species second only to the MAC (41). This complex increasingly leads to opportunistic pulmonary and skin infections (42). Clinically, the treatment effect for it is not ideal, thus demanding sufficient attention. Data in this study indicate that the *M. chelonae/*MABC exhibits a high sensitivity rate of 90.9% to amikacin, showing good susceptibility. However, its sensitivity to linezolid and clarithromycin is relatively low. Hence, before incorporating these two drugs into the treatment regimen, it is advisable to base the selection on the results of AST. Cefoxitin is crucial in treating *M. chelonae/abscessus* infections. Nevertheless, drug susceptibility tests reveal that the complex’s sensitivity to cefoxitin is less than ideal, with a sensitivity rate of merely 36.4%, while 59.1% of cases are at an intermediate level. Therefore, in clinical practice, a high dosage of cefoxitin should be administered when treating *M. chelonae/abscessus* infections (6). It shows high resistance to moxifloxacin, doxycycline, tobramycin, imipenem, and sulfamethoxazole, so empirical application is not recommended. For drugs without clear breakpoints, such as ethambutol and rifamycins, they all show high MIC values, indicating resistance. (iii) The clinical efficacy and prognosis of *M. kansasii* disease are better than those of other mycobacterial diseases, and most cases can be effectively treated with a regimen consisting of rifampicin, isoniazid, and ethambutol in clinical practice (1). The research data show that *M. kansasii* has good sensitivity to clarithromycin, amikacin, moxifloxacin, linezolid, and rifampicin. However, the resistance rates to sulfamethoxazole and doxycycline are relatively high, and empirical use is not recommended. Among the drugs without breakpoints, imipenem and tobramycin both show high MIC values of 100%, indicating resistance. (iv) *M. xenopi* has good sensitivity to clarithromycin, amikacin, moxifloxacin, linezolid, and rifampicin, but poor sensitivity to doxycycline and minocycline. However, the quantity of *M. xenopi* collected this time is small, with only two strains, which has no statistical significance.

The treatment of NTM diseases is difficult and complex. Based on clinical work experience, the following factors are mainly considered: (i) Long treatment cycle and high cost: For NTM pulmonary diseases, the treatment is required to continue for at least 1 year after the sputum culture turns negative, and the total treatment course is about 18 months. Consequently, the treatment cost is high (6). Moreover, the treatment of NTM mainly takes place in pulmonary hospitals or the respiratory departments of large comprehensive hospitals. The lack of convenience in regular examinations and medication dispensing further increases the burden on patients. (ii) Severe drug side effects or inconvenient administration: Combined medication, especially the macrolide drugs clarithromycin or azithromycin, which are the core drugs for treating MAC, can cause severe gastrointestinal reactions (43). In this study, 16 patients did not receive treatment from the beginning due to severe gastrointestinal reactions, and 9 more patients withdrew from the treatment later due to gastrointestinal reactions. This is an inevitable problem in the treatment of NTM diseases. Ethambutol can cause optic neuritis, and rifampicin can lead to liver function damage and leukopenia. The side effects become more obvious with long-term use (43). It is also difficult to use amikacin injection and cefoxitin injection for a long time during treatment. (iii) High recurrence rate: Since NTM widely exists in the surrounding living environment and has close contact with the respiratory tract, if disinfection work is not properly carried out, the possibility of reinfection is very high. Among the cases collected in this study, there were three cases of extrapulmonary NTM infections, all of which participated in the treatment and were cured. Therefore, repeated exposure to NTM in the environment may also be a reason for the high recurrence rate. (iv) NTM diseases are not classified as contagious diseases in China, and there is no mandatory reporting or standardized treatment, along with a lack of effective supervision. In clinical practice, after some patients learn that nontuberculous mycobacteriosis is not a contagious disease, their willingness to receive treatment decreases. Some patients stop the treatment when they feel that their symptoms have improved, or they think the treatment effect is not satisfactory. Some doctors themselves are not proactive in treating NTM diseases, which makes the treatment of NTM diseases full of too many uncertain factors. It is also very difficult to calculate the effective treatment rate.

Finally, the main limitations of this study are the relatively short treatment observation period and the failure to collect more cases, including the treatment situations of patients without MIC drug susceptibility testing, as controls. The sputum examination results were also not fully recorded. In the future, more high-quality studies or controlled studies will be conducted to provide a higher level of evidence for the diagnosis and treatment of NTM and guide clinical practice.

### Conclusion

In the local area, the population affected by NTM diseases is predominantly composed of middle-aged and elderly men. When patients present with chronic cough symptoms and imaging examinations reveal the presence of bronchiectasis and cavity formation, it has a significant effect on the diagnosis of NTM diseases. Regarding the distribution of bacterial species, *M. intracellulare* is the most prevalent in this region, followed by the *M. chelonae/*MABC and *M. avium*. *In vitro* AST demonstrated that MAC exhibits relatively high sensitivity to clarithromycin, amikacin, ethambutol, rifapentine, moxifloxacin, and rifampicin. The *M. chelonae/*MABC is only sensitive to amikacin. *M. kansasii* shows a high degree of sensitivity to clarithromycin, amikacin, moxifloxacin, linezolid, and rifampicin. Whether patients receive anti-NTM treatment is influenced by a combination of multiple factors. Currently, approximately two-thirds of the patients choose to receive anti-NTM treatment. Moreover, research has found that patients can ultimately benefit from adhering to the treatment for more than 6 months.
